# Repulsive expansion dynamics in colony growth and gene expression

**DOI:** 10.1371/journal.pcbi.1008168

**Published:** 2021-03-18

**Authors:** Yangxiaolu Cao, John Neu, Andrew E. Blanchard, Ting Lu, Lingchong You

**Affiliations:** 1 Department of Biomedical Engineering, Duke University, Durham, North Carolina; 2 Computational Sciences and Engineering Division, Oak Ridge National Laboratory, Oak Ridge, Tennessee; 3 Department of Bioengineering, University of Illinois at Urbana-Champaign, Urbana, Illinois; 4 Carl R. Woese Institute for Genomic Biology, University of Illinois at Urbana-Champaign, Urbana, Illinois; 5 Center for Biophysics and Quantitative Biology, University of Illinois at Urbana-Champaign, Urbana, Illinois; 6 Center for Genomic and Computational Biology, Duke University, Durham, North Carolina; 7 Department of Molecular Genetics and Microbiology, Duke University Medical Center, Durham, North Carolina; King’s College London, UNITED KINGDOM

## Abstract

Spatial expansion of a population of cells can arise from growth of microorganisms, plant cells, and mammalian cells. It underlies normal or dysfunctional tissue development, and it can be exploited as the foundation for programming spatial patterns. This expansion is often driven by continuous growth and division of cells within a colony, which in turn pushes the peripheral cells outward. This process generates a repulsion velocity field at each location within the colony. Here we show that this process can be approximated as coarse-grained repulsive-expansion kinetics. This framework enables accurate and efficient simulation of growth and gene expression dynamics in radially symmetric colonies with homogenous z-directional distribution. It is robust even if cells are not spherical and vary in size. The simplicity of the resulting mathematical framework also greatly facilitates generation of mechanistic insights.

## Author summary

Spatiotemporal dynamics are ubiquitous in biology. To understand these phenomena in nature or to program them using synthetic gene circuits, it is critical to resort to mathematical modeling to deduce mechanistic insights or to explore plausible outcomes. Historically, modeling of spatiotemporal dynamics depends on the use of agent-based models or their continuum counterparts consisting of partial differential equations. Here, we show that a class of colony expansion can be treated as being driven by the steric force generated by growing and diving cells. This approximation leads to a drastically simplified framework consisting of only ordinary differential equations. This framework greatly improves the computational efficiency and facilitates development of mechanistic insights into the dynamics of colony growth and pattern formation.

## Introduction

Spatial expansion of a population of cells is ubiquitous in biology. It can arise from growth of bacterial or yeast colonies [1,2], development of plant tissues [3], animal tissues [4], or growth of tumors [5–7]. Understanding the expansion dynamics of a cell population is important, since colony expansion is a reflection of many cellular properties. For example, the substrate nutrient uptake rate, the metabolism, the division and death rate at the individual cell level. In addition, since colony expansion is usually coupled with the gene expression activity and the metabolic activity, understanding the expansion dynamics is the foundation of understanding the spatial dynamics of gene expression in growing colonies [8–11].

To date, primarily two types of models have been used to simulate spatial dynamics in growing colonies (including gene expression): agent-based models (ABMs) and continuum models based on partial differential equations (PDEs). An ABM focuses on simulating a single cell or a cluster of cells as an agent; each agent behaves identically. The overall behavior of the population emerges from the interactions between agents, through contact or diffusible chemicals. Using the ABM entails assigning pre-defined rules and parameters to each agent. It provides detailed information about individual agents, as well as the overall system. However, it has several potential limitations. First, certain ABM requires more extensive assumptions about the definition of each agent, how each behaves, and how different agents interact [12,13]. Many of these assumptions are made based on self-consistency and require parameters that are difficult to measure independently. Second, by construction, the computational demand by an ABM scales with the number of agents, as well as the complexity of reaction kinetics in each agent. As a result, ABMs are typically computationally expensive, making it difficult or even impossible to simulate the spatiotemporal dynamics of a cell population containing tens of millions of cells (a visible bacterial colony contains about 10–100 million cells). Finally, because of the sheer complexity of inter-agent interactions and the computational cost, it is difficult to deduce intuitive understanding of the properties emerging from the overall system [14].

These limitations can be alleviated by using continuum models consisting of PDEs. However, numerically solving PDEs can also be computationally demanding (though typically less so than ABMs) [15]. Even though these tools can generate simulation results that can recapture certain aspects of the experimental results, it is difficult to draw mechanistic understandings from the simulation.

In general, the colony expansion can be driven by a combination of internal and external forces. For instance, cells can preferentially move toward certain environmental cues, for example, by chemotaxis [16–18]. They can also modulate motility by secreting and responding to surfactants, resulting in swarming [19–26]. Irregular colony morphology can result from buckling and hierarchical wrinkling [10].

In many cases, however, the expansion is primarily driven by continuous growth and division of cells in the colony [27]. That is, the cells in the interior of a colony collectively push the peripheral cells outward, leading to colony expansion. The driver is the mechanical force generated by growth. This notion has been well recognized in diverse organisms, including bacteria [11,28,29], yeast [30], plant cells [31,32], and tumor cells [33,34]. Recognizing this feature enables us to model the colony expansion and gene expression using a highly simplified framework. By reducing computational complexity, this framework drastically accelerates numerical simulations and facilitates development of mechanistic insights into the underlying dynamics of interest.

## Results

### The repulsive expansion model captures the colony growth dynamics

**Fig 1:**
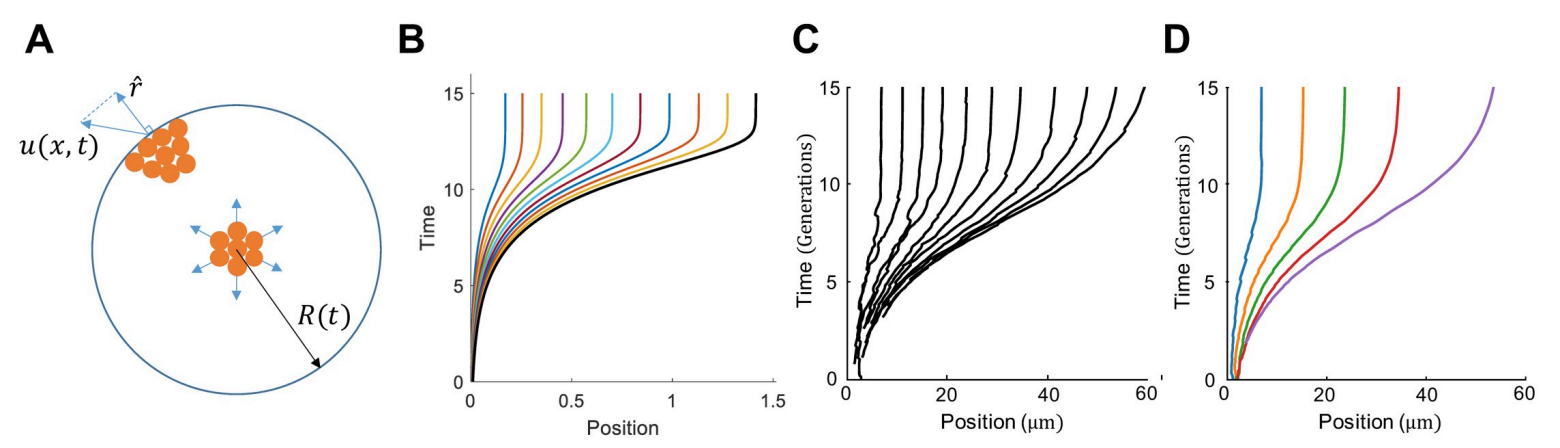
The repulsive expansion model captures colony growth dynamics. A. Illustration of the repulsive expansion dynamics. Each orange dot represents a cell at that given location. As cells in the colony grow and divide, cells in the interior will push the outer cells, generating a velocity field *u*(*x*,*t*). Given a location, $\hat{r}$ is the normal vector along radial direction. Therefore $u∙\hat{r}$ represents the radial expansion velocity at the location. Since the model is assumed to be radial symmetric, the most outer layer of cells determines the size of the colony. At time *t*, the colony radius is expressed as *R*(*t*). The different color lines indicate different cell trajectories. Colors are chosen simply for distinguishable visualization effect. B. Colony growth over time. The black line indicates the radius of the colony, the colored lines represent the trajectories of pre-selected 10 locations within the colony. C. Sample trajectories of individual cells in the ABM simulation. Each trajectory shows the movement of a single cell. Here, cells are approximately spherical; time is measured in generations (~30 minutes). D. Average trajectories of cells in the ABM simulation over time. Cells were assigned to 5 different groups according to their radial positions when the total population of the colony reached 1000 cells; subsequently, all the 1000 cells were tracked over time in terms of their radial positions. Each of the colored lines shows the average trajectory of the cells in each group.

To develop our framework, we first assume the steric force between cell-cell growth and division are the driving force of colony expansion. At a particular location, the local steric force will generate a steric repulsion velocity field. We then assume that cells are spheres with the same radius; therefore, on average, the overall pushing force on each cell is perpendicular to the boundary of a radially symmetric colony. If the profile of this velocity field is known with given initial conditions, one can use sets of ODEs to simulate the moving trajectories of any objects within the colony (Fig 1A).

In a close-packed colony, *σ*(*x*, *t*) denote the cell division rate at position *x* and time *t*. The cell division rate depends on nutrient concentration *n*(*t*), and the relative position within the colony. Here, we assume that the nutrient concentration is uniformly distributed, which is applicable when the nutrient diffusion length scale is much larger than the colony size. Thus, its concentration is only a function of time, *n*(*t*). Therefore, *σ*(*x*, *t*) can be expressed as:

$$\sigma (x,t)=\sigma_{0}\frac{n(t)}{n(t)+n_{*}}\frac{{K_{\sigma }}^{n_{\sigma }}}{{K_{\sigma }}^{n_{\sigma }}+{\left(R(t)-x\right)}^{n_{\sigma }}}$$ (1)

The rate of change in the colony volume can either be expressed as a function of the normal velocity of the colony boundary or the total number of the cells. The velocity field of given each position could be expressed as a function of cell division rate and the relative position within the colony (see S1 Text for detailed derivations). Here, *v* is the volume per cell, $\tilde{\sigma_{n}}$ is a parameter related with maximum nutrient consumption rate *σ*_*n*_

$$\left\{ \begin{array}{c} \dot{r}=\frac{v}{r}\int_{0}^{r}x\sigma (x,t)dx\\ \dot{R}=\frac{v}{R}\int_{0}^{R}x\sigma (x,t)dx\\ \dot{n}=-\tilde{\sigma_{n}}\int_{0}^{R}x\sigma (x,t)dx \end{array}\right.$$ (2)

Numerically solving these equations (see S1 Table for the parameter values and the numerical procedure) can generate the dynamics of colony expansion as a function of time. Fig 1B shows a typical set of results on colony radius expansion (solid black line) and the trajectories within the colony (colored lines). Each trajectory indicates how a cell would move if its position is in the trajectory. This notion could be counter-intuitive if a cell does not exist initially and thus does not have a location in the initial colony. For example, consider a cell born at location *r*_1_ and at time *t*_1_. If a velocity field trajectory passes this position (*r*_1_, *t*_1_), the trajectory would predict how the cell would move at any time point later than *t*_1_. The section of the trajectory before *t*_1_ would be imaginary. However, to simplify presentation, all the trajectories are plotted from time zero in our simulation.

To evaluate the validity of the fundamental conceptual framework of the repulsive expansion model, we still used an ABM [35] to simulate colony growth with similar physical constraints. In brief, rigid rods surrounded by deformable shells serve as agents that represent individual cells. Mechanical forces are calculated by determining the overlap between spheres placed at the closest points between cells. Each agent can elongate and divide upon the consumption of diffusive nutrient, mimicking cellular growth and division processes. Such a modeling scheme enables simulations of colony expansion in an individual and mechanical manner [35]. A brief description of the equations and parameters governing cell growth and nutrient diffusion is provided in S2 and S3 Tables. A full description of parameters and equations for the ABM can be found in previous work [35].

For cells with an approximately spherical shape, Fig 1C shows sample trajectories of single cells as the colony grows from 10 to 30,000 cells. Fig 1D, on the other hand, depicts the averaged movement of the cells in the process. Consistent with the repulsive expansion model, both the sample and average trajectories increase initially and, over time, settle to constant values due to nutrient decline for the interior of the colony.

**Fig 2:**
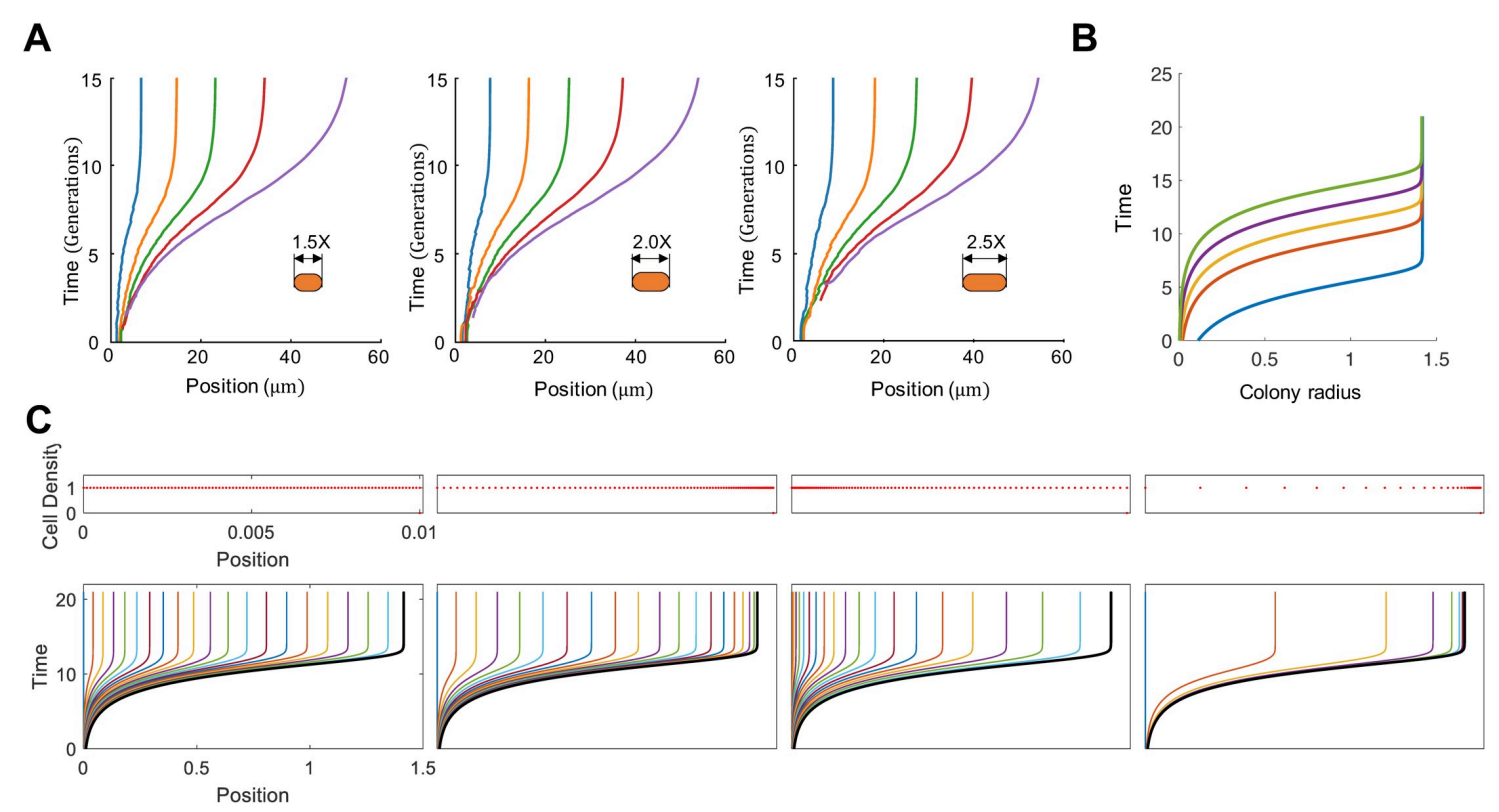
The repulsive expansion model under different initial model settings. A. Average trajectories of cells in the ABM simulation with different length-to-width ratios. Similar to Fig 1D, cells were assigned to 5 different groups according to their radial positions when the total population reached 1000 cells and, subsequently, tracked over time in terms of their radial positions. The left, middle and right panels correspond to the length-to-width ratios of 1.5:1, 2:1 and 2.5:1 respectively. The trajectories show similar behaviors despite the variation of aspect ratio. B. Simulated colony growth using the repulsive expansion model, assuming different initial colony sizes. The top panel shows four cell density distribution with the same shape, the same initial selected positions, but different initial colony size. The bottom panel shows the colony radius expansion over time with different initial colony size (same color code). C. Simulated colony growth using the repulsive expansion model, assuming different initial cell-density distributions. The top panel shows four cell density distributions with the same initial colony size and the same initial selected positions. The bottom panel shows the colony radius expansion over time with different cell density distribution shape (same color code). Each subfigure in the panel has the same x-axis and y-axis. For visual clarity, the axis label and title were shown only in the left most subfigure.

While the repulsive expansion model is derived assuming spherical cells, it remains to be reliable even when this assumption is relaxed, as validated by the ABM simulation (Figs 2A and S1). Similar to the approximately spherical cells, the average trajectories of the cells with different length-to-width ratios continue to show a similar radius expansion pattern that involves an initial increase and a gradual approach to constant values. This consistency indicates that the heterogeneity in cell size and shape is averaged out during colony growth and expansion, making the repulsive expansion model a reliable simplification. The colony expansion dynamic from repulsive expansion model is comparable to the results solved from PDEs or ABM. For example, using repulsive expansion model (see S1 Table for parameter values), regardless of the initial colony size, with the same environmental condition, the final colony sizes are the same (Fig 2B).

An important advantage of the repulsive expansion model is that the accuracy of computation does not depend on the resolution of the spatial discretization in initiating the simulation. This property drastically increases the computation efficiency without sacrificing computational accuracy. In Fig 2C, despite the segmentation of the spatial meshes, given the same initial colony size, the moving trajectories within the colony are the same (Fig 2C). This property does not occur in the ABMs or PDE models, where a sufficiently high resolution in discretizing the space is critical to ensure computational accuracy or reliability. However, in the repulsive expansion model, a cell trajectory is directly determined by cell growth rate (Eq 2). Once the cell growth rate is given, the trajectory of each position could be computed indepdent of other trajectories, which does not require fine-grained space discretization to achieve high computational accuracy. This feature will substantially simplify the simulation of cell movements.

### Modeling programmed pattern formation dynamics using the repulsive expansion model

**Fig 3:**
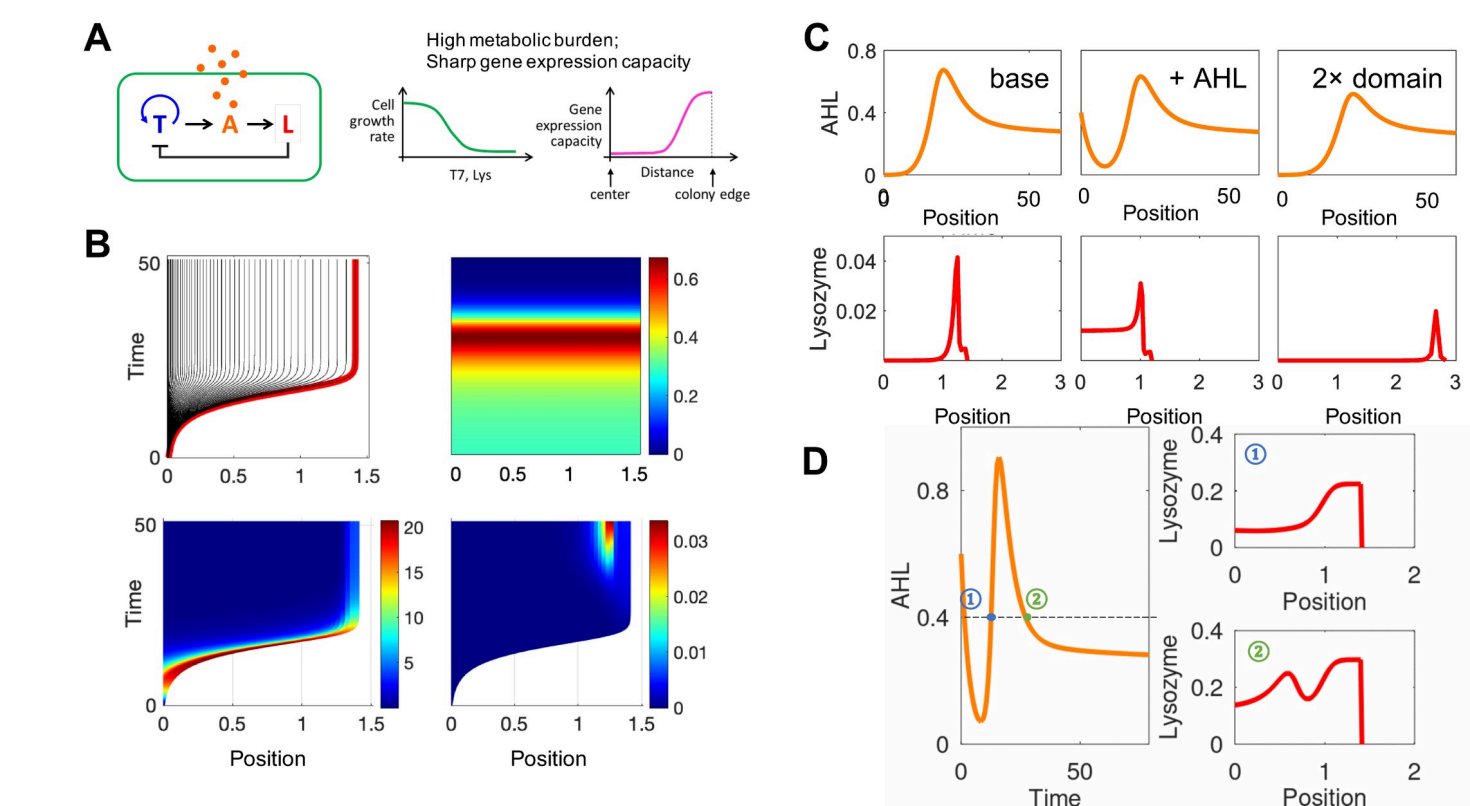
Simulated spatial-temporal dynamics of the pattern formation in engineered bacteria with a high metabolic burden and a sharp gene expression capacity profile. A. Left: circuit logic. Middle: the model was simulated under the condition of high metabolic burden. The cell growth rate is a decaying function of the production of T7 RNAP and T7 lysozyme. Right: Gene expression capacity. The x-axis represents the distance from the colony edge. B. Top left to bottom right: Simulated spatial-temporal dynamics of colony radius, AHL, T7 RNAP and T7 lysozyme for varying distance (x-axis) over time (y-axis), respectively. The parameters used in the simulation are listed in S4 Table. C. Simulated pattern formation with different environmental factors. The top panels from left to right shows the AHL dynamic under the base case (left, same with (B)), with initial AHL concentration is 0.3 (middle), and with domain size which is twice as large as the base case (right). The bottom panels are the T7 lysozyme distribution at the time when nutrient is exhausted under each condition, respectively. The x axis represents the distance from the colony edge. D. Simulated double rings. Left: the AHL dynamic with initial AHL concentration 0.8. Right top panel: the lysozyme distribution at time point 1, which is labeled in the left panel. Right bottom panel: the lysozyme distribution at time point 2.

The repulsive expansion model can be extended to describe gene expression dynamics that are coupled with colony expansion. To demonstrate this practice, we apply this framework to the analysis of a synthetic pattern-formation circuit that we recently engineered [8,9]. The circuit consists of a T7 RNA polymerase (T7) that activates its expression. Upon activation by T7, synthesis of AHL (A) will be mediated, which can diffuse across the cell membrane. When the global AHL concentration surpasses a threshold, intracellular AHL will trigger the activation of the synthesis of T7 lysozyme (L). Lysozyme then binds to the T7 and forms a T7-lysozyme complex (P), therefore inhibiting the T7 binding to the T7 promoter. This T7-lysozyme complex also inhibits T7 transcription [36]. In this process, the AHL concentration is affected by its initial concentration and the domain size. The expression rates of T7, lysozyme, and AHL are all controlled by the spatially dependent gene expression capacity (Fig 3A).

Depending on the experimental conditions, including cell strains and growth substrates, the circuit can generate different patterns. In particular, Payne et al. demonstrated the generation of one sharp ring or multiple rings when starting from single bacteria within a semi-solid agar droplet. The generation of robust ring pattern is without apparent morphogen gradient. The morphogen serves as a timing cue, can trigger multiple or scalable ring patterns. Cao et al. demonstrated the generation of core-ring patterns that scale with the colony size when initiating the growth and patterning process from a few cells inkjet-printed to the top of agar surface. In both cases, the models are assumed to be radial symmetry. One study is in a one-dimensional ABM [8] and one used a PDE model [9].

**Fig 4:**
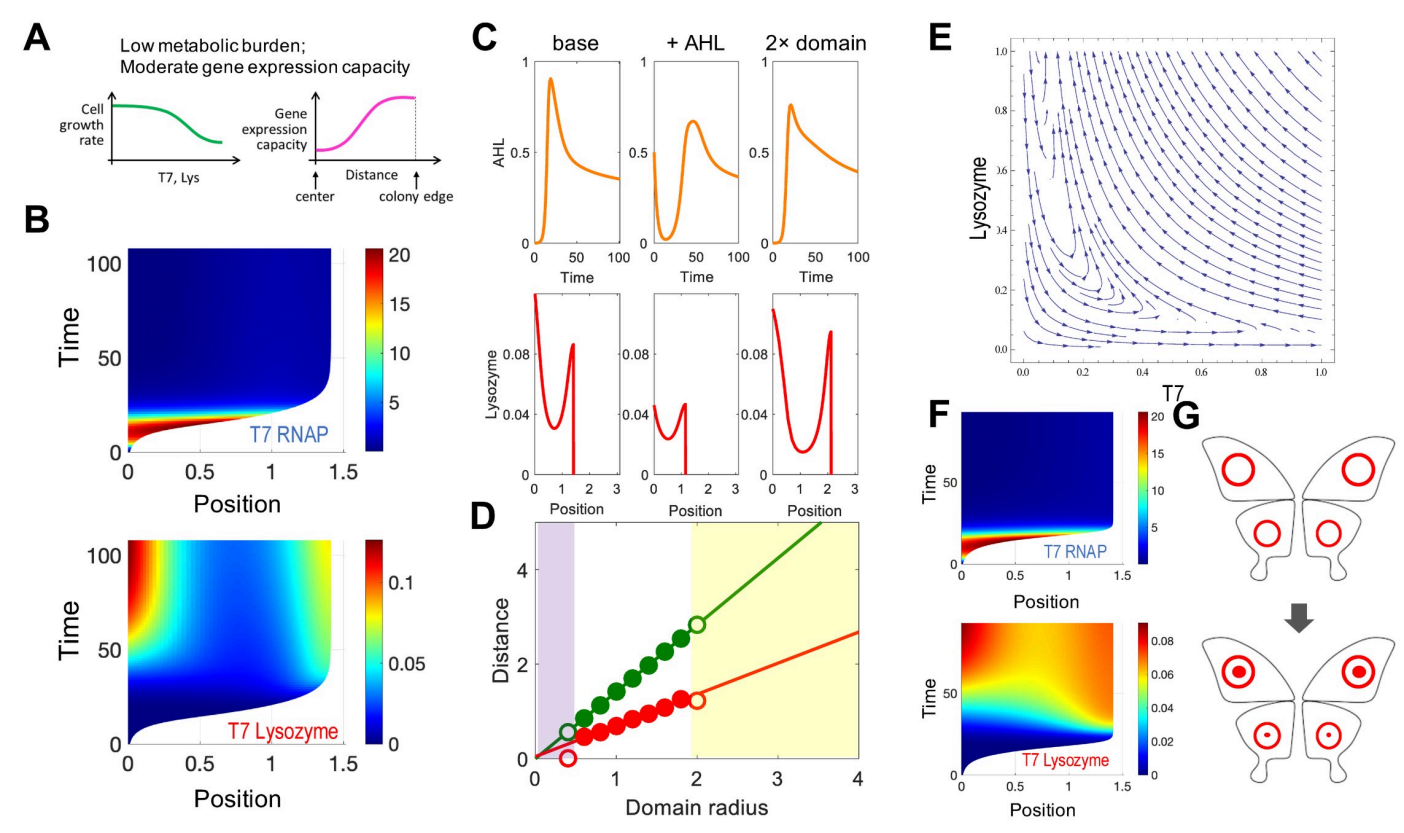
Simulated spatial-temporal dynamics of the pattern formation process with a high metabolic burden and a flat gene expression capacity profile. A. Simulation assumption with a low metabolic burden and a moderate gene expression capacity. The notations are the same with Fig 3. B. Simulated spatial-temporal dynamics of T7 RNAP and T7 lysozyme for varying distance (x-axis) over time (y-axis). The parameters used in the simulation are listed in S4 Table, except *K*_*σ*_ = 0.1; *n*_*σ*_ = 2; *n*_*φ*_ = 2; *K*_*φ*_ = 0.1. C. Simulated pattern formation with different environmental factors. The top panels from left to right shows the AHL dynamic under the base case (left), with initial AHL concentration 0.3 (middle), and with domain size which is twice as large as base case (right). The bottom panels are the T7 lysozyme distribution under each condition, respectively. The x-axis represents the distance from the colony edge. D. Simulated scale invariance in pattern formation. Dependence of the ring width (red circles) and the colony radius (green circles) on the domain radius from 1 to 4. The lines represent the linear regression of the colony radius and the ring width with respect to the domain radius in the white region. E. Phase diagram of T7 lysozyme (y axis) to T7 RNAP (x axis). Based on the phase plane diagram, given an initial condition (T7RNAP = 0.1, T7 lysozyme = 0), T7 RNAP increases first then decreases, while T7 lysozyme keeps increasing. F. Simulated spatial-temporal dynamics of T7 RNAP and T7 lysozyme for varying distance (x-axis) over time (y-axis). The parameters used in the simulation are listed in the S4 Table, except *K*_*σ*_ = 0.1; *n*_*σ*_ = 2. G. Ring pattern formation in the butterfly wings. Since eyespot signals to from inner and outer rings are released at different time-points. The outer rings form first, then the inner rings form within the outer rings at a later time point.

Using comparable settings, the ABM was most time consuming–with an average simulation taking ~10hrs. The PDE model drastically improved the efficiency by reducing the time to ~20min. With appropriate parameter choices, the PDE model can also reproduce the qualitative aspects of the outcomes from the ABM. The circuit dynamics can be readily implemented using the repulsive-expansion model (Eq 12 in S1 Text), which generates similar patterns comparable to the ABM (Fig 3B) or PDE (Fig 4B) models but with a speed that is 18,000-fold faster than the ABM model and 170-fold faster than the PDE model.

Similar to the analysis done in Cao et al, choosing appropriate parameters can allow us to generate the different patterns observed in both studies. When changing the environmental factors, the repulsive expansion model successfully recaptures the dynamics reported in Payne et al. Given an initial AHL concentration, a ring with a smaller radius forms around the colony edge compared to the one in the base case (without initial AHL). Given a larger initial domain size, a ring with a larger radius will form around the edge of a larger colony (Fig 3C). These results are consistent with the experimental and computational results presented in Payne et al. Two lysozyme rings can form given a high initial AHL concentration, which is consistent with the observations in Payne et al (Fig 3D).

In the study by Cao et al., with an initial AHL concentration, a core and a ring with narrower width formed near the edge of the colony compared to the base case (without initial AHL). Given an initial larger domain, the repulsive expansion model indeed generates a wider core and ring will form in a larger colony (Fig 4C). Moreover, the simple model also successfully generates the scale invariance reported in Cao et al: the ring width and the colony radius are both proportional to the domain radius, when the latter is of moderate magnitude (Fig 4D).

In addition to the superior computational efficiency, the simplicity of the modeling framework provides more intuitive insights of circuit dynamics. For example, Payne et al. and Cao et al. described two types of lysozyme patterns using the same circuit. Since the repulsive expansion model is simpler and with fewer parameters, it is easier to test how each parameter affects the system’s dynamics. In this case, the level of metabolic burden and gene expression capacity are two important factors to generate different types of patterns, which indicates the temporal cooperation between colony growth and gene expression are crucial for forming different types of patterns.

As another example, the dynamics of T7 is observed to be faster and more transient than lysozyme’s (Fig 4B). In Cao et al., the gene expression dynamics and the cellular movements are coupled in the PDEs. However, in the repulsive expansion model, particularly when the metabolic burden is small, the colony expansion could be modeled as independent of circuit dynamics (Fig 4A). By separating the cellular movement from gene expression, we can simplify the model into two layers: the trajectories of cells that reflect cellular movement, and the gene expression dynamic along each trajectory. In the phase diagram of T7 and lysozyme, when T7 and lysozyme all start with small initial values, T7 will increase to a peak then decrease, while the significant accumulation of lysozyme happens after the peak of the T7 (Fig 4E). By combining these gene expression dynamics with the trajectories of cells within the colony, we can see this correlation between the T7 patterns and lysozyme patterns. Compared with the ABM or the PDE model, the repulsive expansion model provides more intuition when it is used to analyze the dynamics of spatial patterns.

Since the repulsive expansion model describes cell moving trajectory and gene expression separately, this framework also provides direct insights into the relationship between cellular movement and circuit dynamics. In the previous PDE model, there are in total 22 parameters, which is very high dimensional. To reduce the complexity, the parameters that dictate to colony growth were fitted to experimental data. Cao et al. concluded the T7 lysozyme profile is mainly determined by circuit logic and growth dilution. During the colony expansion, near the colony edge, the lysozyme is insufficient to overcome the dilution of the cell growth. Therefore, a strong lysozyme core occurred before the ring formation.

The previous study has focused on the patterning process when the cell division and expansion rates are high. Given the simplicity of the repulsive expansion model, we can readily test the effects of the cell division and expansion rate when they are small. Interesting, our new modeling result predicts the core-ring pattern formation with the ring forming before the core (Fig 4F). Lysozyme accumulates near the colony edge due to the high gene expression capacity near the colony edge, which leads to ring formation. However, due to the circuit dynamic, the accumulating T7 and AHL lead to lysozyme increasing near the center of the colony. This patterning process of outer ring develops earlier than the inner core is analogous to the typical eyespot pattern-formation process in butterfly wings. On the background of wings, there are a layer of parafocal elements (PFEs), which serve as the pattern units. The eyespot patterns are the PFEs with different color distributions. Many studies have shown the eyespot signals that form inner and outer rings are released at different time-points. The outer ring forms before the inner ring (Fig 4G) [37,38]. The network and the parameter combination identified by the repulsive expansion model could provide insights of how eyespot patterns are formed in butterfly wings.

## Discussion

The repulsive expansion model models the “flow” of cells in a growing colony, driving by the force generated by cell growth and division. One can image of a perfect radial symmetric colony, given the nutrient concentration and distribution, the colony growth rate of each cell at any position is known. Therefore, the trajectory of any cell with given initial position can be calculated. It offers superior computational efficiency as it does not require high resolution of space discretization to ensure computational accuracy. The framework is readily amendable to the modeling of spatial temporal dynamics coupled with or arising from colony growth and expansion. We have illustrated this point by applying the modeling framework to a synthetic gene circuit that has been previously analyzed.

In general, the framework could be applied to a class of spatial distribution problems: the system is radially symmetric, with homogenous z-directional distribution, and with gradient-free chemicals or chemicals that have gradient but exceeds the triggering threshold. These criteria are the cornerstone of the repulsive expansion dynamics. Many examples of biological processes indeed satisfy these conditions. Examples include colony growth and gene expression in a microfluidic chip with a confined chamber height [39–41], a single layer of cell divisions in a microscope slide [42–44], and cells in 3D symmetric spherical growth condition [45,46]. If these systems can secrete chemicals as global signaling molecules, the extra requirement is that the signaling molecules’ diffusion rates are fast enough to form uniform spatial distributions [8,9], or the molecules accumulate fast enough to exceed beyond the gene expression triggering threshold [47,48]. If the chemical gradients significantly affect the colony expansion and expression, our modeling framework can be extended to incoporate such gradients as long as radial symmetry is maintained. In particular, this extension can be achieved by adjustifying time-dependent parameters (e.g. cell division rate) to reflect the effects of chemical gradients. Moreover, heterogenous cell-cell interactions [49] can affect colony expansion dynamics and cause additional deviations between the predictions of the simple model and experiment or more comprehensive models.

## Supporting information

S1 Text Details on the derivation of the ODE model. (PDF)

S1 Fig Sample trajectories for cells in ABM with different aspect ratios. (PDF)

S1 Table Definitions and values of parameters used in the ODE model (colony growth). (PDF)

S2 Table Growth and nutrient equations in ABM. (PDF)

S3 Table Parameters used in the ABM. (PDF)

S4 Table Definitions and values of parameters used in the ODE model (patterning). (PDF)
