# Supplementary material for: Repulsive expansion dynamics in colony growth and gene expression
Source: PLoS Comput Biol. 2021 Mar 18;17(3):e1008168. doi: 10.1371/journal.pcbi.1008168 (PMC8009408; doi:10.1371/journal.pcbi.1008168)
Supplement: S1 Text — (PDF) [file pcbi.1008168.s001.pdf]

S1 Text
Details on the derivation of the ODE model

Construction of the repulsive expansion model to simulate the colony growth

In a close-packed colony, let V denote the area of the colony and $\sigma(x,t)$ denote the cell division rate at position x and time t. The cell division rate depends on nutrient concentration $n(t)$, and the relative position within the colony. Here, we assume that the nutrient concentration is uniformly distributed; thus, its concentration is only a function of time, $n(t)$. Therefore, $\sigma(x,t)$ can be expressed as:

$$\sigma(x,t) = \sigma_0 \frac{n(t)}{n(t)+n_*} \frac{{K_\sigma}^{n_\sigma}}{{K_\sigma}^{n_\sigma}+(R(t)-x)^{n_\sigma}}, \quad when\, x \leq R(t) \tag{1}$$

where σ_0 is the maximum division rate, n_* reflects the sensitivity of σ on $n(t)$. The 2nd Hill term indicates the decreasing division rate as the distance ($R(t) - x$) from the edge of the colony, which has a position $R(t)$. The extent by which this dependence is nonlinear is tuned by parameters K_σ and n_σ.

In each colony, the collective growth and division of cells creates a steric force that "pushes" peripheral cells outwards, generating a velocity field $u(x,t)$ inside the colony. Let $S(t)$ be a region whose boundary $\partial S(t)$ moves with the cells, as depicted in Figure 1A. The region $S(t)$ is a circle with radius $R(t)$. The normal velocity of the boundary is $u \cdot \hat{r}$ Thus, the rate of change in the $V(t)$ of the entire colony follows:

$$\dot{V} = \oint_{\partial S} u \cdot \hat{r}\, dx = \iint_S \nabla \cdot u\, dS \tag{2}$$

The second step of the equation applies the Green's theorem. Alternatively, the total volume of the colony is proportional to the total number of cells, $N(t)$ and the volume per cell, v.

$$V = vN\,(t) \tag{3}$$

The rate of change in $N(t)$ is determined by cell division rate throughout the region $S(t)$.

$$\dot{N} = \iint_S \sigma(x,t)\, dS \tag{4}$$

Combining Equations (3) and (4) leads to:

$$\dot{V} = \iint_S v\sigma(x,t)\, dS \tag{5}$$

Equations (2) and (5) are applicable to all regions $S(t)$, which requires:

$$\nabla \cdot u = v\sigma(x,t) \tag{6}$$

inside the colony.

In the simplest situation of a radially symmetric colony, the unit vector along is along the radial direction. Therefore, the radial velocity field at radius r and time t can be expressed as $u = u(r,t) \cdot \hat{r}$. Therefore, $u(r,t)$ satisfies:

$$\nabla \cdot u = u_r + \frac{u}{r} = \frac{\partial(ur)}{r\partial r} = v\sigma(x,t) \tag{7}$$

Given the initial condition $u = 0$ at $r = 0$, the unique solution of equation (7) is:

$$u = \frac{v}{r}\int_0^r x\sigma(x,t)dx, 0 \le r \le R \tag{8}$$

Now we introduce the cell trajectories, $r = r(t)$. Once a cell is born at radius r_0, the trajectory satisfies the initial value problem:

$$\frac{d}{dt}r(t) = u(r(t),t)\cdot \hat{r} = \frac{v}{r(t)}\int_0^r x\sigma(x,t)dx, t \ge 0, r(0) = r_0 \tag{9}$$

The overall nutrient consumption rate is determined by the nutrient consumption rate per unit cell density σ_n, and the nutrient concentration $n(t)$. If the volume of the agar is Ω, based on the simple conservation law:

$$n(t) = n(0) - \frac{\sigma_n}{\Omega v}\pi R(t)^2 \tag{10}$$

For simplicity, we define $\widetilde{\sigma_n} = \frac{2\pi\sigma_n}{\Omega v}$. Taken together, the colony expansion can be described by the following ODEs (**the repulsive expansion model**).

$$\begin{cases} \dot{r} = \frac{v}{r}\int_0^r x\,\sigma(x,t)dx \\ \dot{R} = \frac{v}{R}\int_0^R x\,\sigma(x,t)dx \\ \dot{n} = -\widetilde{\sigma_n}\int_0^R x\sigma(x,t)dx \end{cases} \tag{11}$$

Numerically, the equations are solved according to the following initial condition:

- $R(0) = R_0$ for the colony,
- $r(0) = r_0 \le R_0$ for each trajectory. Typically, multiple trajectories are computed when computing the colony growth and expansion.
- $n(0) = n_0$

R_0 defines the initial inoculum size and $R(t)$ reflects the colony size change over time. r_0 represents the initial position of an arbitrary trajectory initiating within the colony. As numerical accuracy of computed trajectory is independent of the number of trajectories and the distribution of their initial positions, the colony expansion can be calculated accurately without fine-grained discretization of space.

The repulsive expansion model simulates dynamics of gene expression coupled with colony growth

Cao et al. simulated colony growth and gene circuit dynamics in a PDE model (1). Since we treat each cell as a moving object. In continuum mechanics, the material derivative computes the time rate of change of velocity for a portion of a material moving with a velocity, $u = u(r, t) \cdot \hat{r}$. It can be expressed as $\frac{D()}{Dt} = \frac{\partial()}{\partial t} + u \cdot \nabla(\,)$;

$$\begin{cases} \dot{r} = \frac{v}{r}\int_0^r x\,\sigma dx \\ \dot{R} = \frac{v}{R}\int_0^R x\,\sigma dx \\ \dot{n} = -\widetilde{\sigma_n}\int_0^R x\sigma dx \\ \frac{DP}{Dt} = \frac{\partial P}{\partial t} + u \cdot \nabla P = k_{LT}TL - d_P P \\ \frac{DT}{Dt} = \frac{\partial T}{\partial t} + u \cdot \nabla T = k_T \frac{T}{T+T_*}\frac{P}{P+P_*}\frac{n}{n+n_*}\varphi(x) - \frac{DP}{Dt} - d_T T \\ \frac{DL}{Dt} = \frac{\partial L}{\partial t} + u \cdot \nabla L = k_L \frac{T}{T+T_*}\frac{a^m}{a^m + a_*{}^m}\frac{n}{n+n_*}\varphi(x) - \frac{DP}{Dt} - d_L L \\ \frac{\partial a}{\partial t} = k_a \int_0^R x \frac{T}{T+T_*}\frac{P}{P+P_*}\frac{n}{n+n_*}\varphi(x)dx - d_a a \end{cases} \tag{12}$$

Here, the gene expression capacity is defined as:

$$\varphi(x) = \frac{K_\varphi^{n_\varphi}}{K_\varphi^{n_\varphi} + (R(t) - x)^{n_\varphi}}, \qquad when\ x \le R(t) \tag{13}$$

where K_φ and n_φ determine the dependence of the gene expression capacity on the spatial location of a cell in the colony (1). This dependence is based on the empirical observation in our experimental systems.

Numerically, the equations are solved according to the following initial condition

- $R(0) = R_0$ for the colony,
- $r(0) = r_0 \le R_0$ for each trajectory. Typically, multiple trajectories are computed when computing the colony growth and expansion.
- $n(0) = n_0$
- $T(0) = T_0, L(0) = L_0$ for each trajectory.
- $a(0) = a_0$

The first three equations of Equations (12) are solved in the same manner as Equations (11). $\frac{D()}{Dt}$ equations can be solved directly along the trajectory with r.

Code availability

The repulsive expansion model is implemented in Matlab. The source code is available at:

https://github.com/youlab/Repulsive-expansion-model

References:

1. Cao Y, Ryser MD, Payne S, Li B, Rao C V., You L. Collective Space-Sensing Coordinates Pattern Scaling in Engineered Bacteria. Cell. 2016;165(3):620–30.
2. Blanchard AE, Lu T. Bacterial social interactions drive the emergence of differential spatial colony structures. BMC Systems Biology. 2015;
3. Collins CH, Leadbetter JR, Arnold FH. Dual selection enhances the signaling specificity of a variant of the quorum-sensing transcriptional activator LuxR. Nature Biotechnology. 2006;
4. You L, Cox RS, Weiss R, Arnold FH. Programmed population control by cell-cell communication and regulated killing. Nature. 2004;
5. Payne S, Li B, Cao Y, Schaeffer D, Ryser MD, You L. Temporal control of self-organized pattern formation without morphogen gradients in bacteria. Molecular Systems Biology. 2013;9(1).
6. Kumar A, Patel SS. Inhibition of T7 RNA polymerase: Transcription initiation and transition from initiation to elongation are inhibited by T7 lysozyme via a ternary complex with RNA polymerase and promoter DNA. Biochemistry. 1997;
