# Supplementary figures and images for: Repulsive expansion dynamics in colony growth and gene expression
Source: PLoS Comput Biol. 2021 Mar 18;17(3):e1008168. doi: 10.1371/journal.pcbi.1008168 (PMC8009408; doi:10.1371/journal.pcbi.1008168)

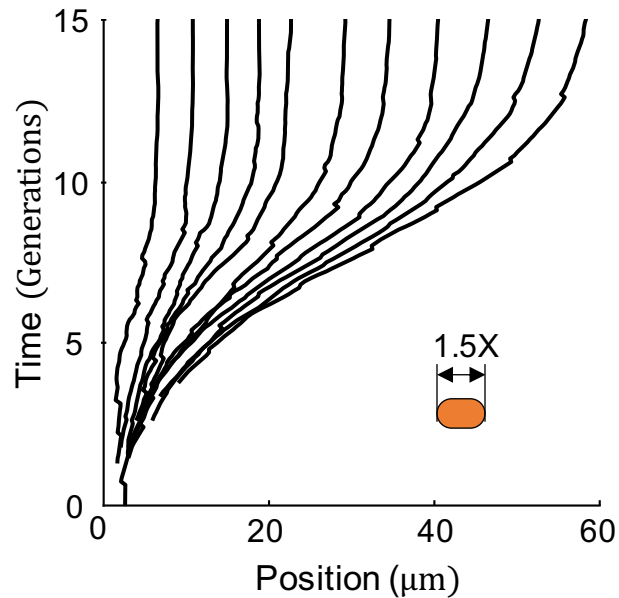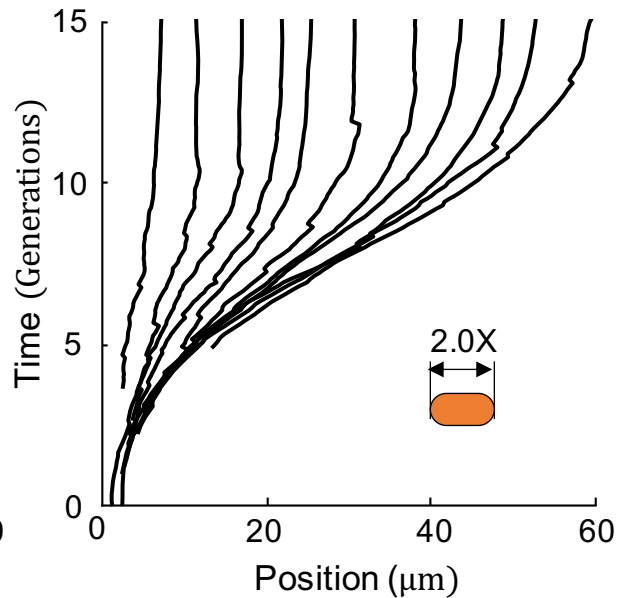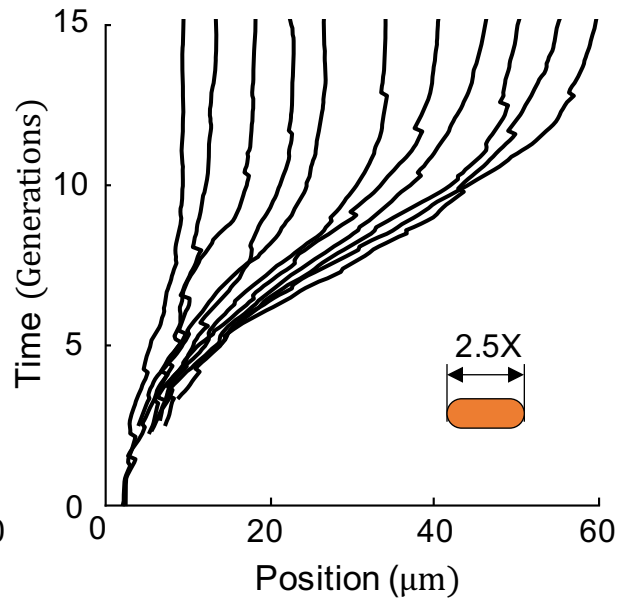

Supplement: S1 Fig — (PDF) [file pcbi.1008168.s002.pdf]
