# Supplementary material for: Repulsive expansion dynamics in colony growth and gene expression
Source: PLoS Comput Biol. 2021 Mar 18;17(3):e1008168. doi: 10.1371/journal.pcbi.1008168 (PMC8009408; doi:10.1371/journal.pcbi.1008168)
Supplement: S1 Table — (PDF) [file pcbi.1008168.s003.pdf]

S1 Table. Definition and the value of parameters used in the ODE model (colony growth)

Parameter	Description	Value	Base Unit
v	volume per unit cell	1	μL/cell
σ_0	maximum cell division rate	1	cell/s/ μL
K_σ	half activation distance for cell division	0.8	mm
n_σ	Hill coefficient for distance-dependent cell division	4	-
R_0	Initial colony radius	0.01	mm
σ_n	maximum nutrient consumption rate per unit cell	27	NU·mm/cell
n_*	half depletion nutrient concentration	0.2	NU/ μL
N_0	Initial nutrient concentration	1	NU/ μL
Ω	agar volume	170	μL
$\widetilde{\sigma_n}$	$\frac{2\pi\sigma_n}{\Omega v}$	1	NU/mm^2/ μL/s

* Nutrient unit (NU) represents a unit amount of the nutrient. The initial amount of the nutrient in the system is 1 NU.
