# Supplementary material for: Repulsive expansion dynamics in colony growth and gene expression
Source: PLoS Comput Biol. 2021 Mar 18;17(3):e1008168. doi: 10.1371/journal.pcbi.1008168 (PMC8009408; doi:10.1371/journal.pcbi.1008168)
Supplement: S2 Table — (PDF) [file pcbi.1008168.s004.pdf]

S2 Table. Growth and nutrient equations in ABM. A complete list of equations is provided in Blanchard et al, reference (2) **in the S1 Text. Here $\boldsymbol{\rho}$ is the area fraction occupied by cells and $\boldsymbol{l_i}$ is the length of a given cell.**

Nutrient (*n*) diffusion	$\frac{dn}{dt} = D_n \nabla^2 n - \alpha_n \frac{\rho n}{\kappa + n}$
Cellular growth	$\frac{dl_i}{dt} = (l_i + 1) \frac{n}{\kappa + n}$
