# Supplementary material for: Repulsive expansion dynamics in colony growth and gene expression
Source: PLoS Comput Biol. 2021 Mar 18;17(3):e1008168. doi: 10.1371/journal.pcbi.1008168 (PMC8009408; doi:10.1371/journal.pcbi.1008168)
Supplement: S3 Table — (PDF) [file pcbi.1008168.s005.pdf]

S3 Table. Parameters used in the ABM. A complete list of parameters is provided in Blanchard et al, reference (2) **in S1 Text.**

Description	Values
Mean length-to-width ratio after division (includes rod length and padding)	1.015 (Fig 1C-D), 1.5 (Fig 2A), 2.0 (Fig 2A), 2.5 (Fig 2A) μm
Fraction of each daughter cell	0.5
Nutrient diffusion grid size	5.0 (for ratios 1.015, 1.5, 2.0), 5.01 (for ratio 2.5) μm
Number of diffusion grids	200
Nutrient diffusion (D_n)	500 $\mu m^2/hr$
Nutrient consumption (α_n)	2 hr^{-1}
Nutrient sensitivity (κ)	0.333
