# Supplementary material for: Repulsive expansion dynamics in colony growth and gene expression
Source: PLoS Comput Biol. 2021 Mar 18;17(3):e1008168. doi: 10.1371/journal.pcbi.1008168 (PMC8009408; doi:10.1371/journal.pcbi.1008168)
Supplement: S4 Table — (PDF) [file pcbi.1008168.s006.pdf]

S4 Table. Definition and the value of parameters used in the ODE model (patterning)

Parameter	Description	Value	Base Unit	Reference	Values in Figure3B	Values in Figure4B
a_*	Concentration threshold of AHL to half-maximum of the pLuxI promoter	20	nM	(3)	20	20
n_σ	Hill coefficient for distance-dependent growth	1			1	4
K_σ	Half activation distance for growth	0.8583			0.8583	0.1
k_a	AHL synthesis rate	9600	molecule $\cdot h^{-1} \cdot cell^{-1}$	(4)	9600	9600
d_a	AHL degradation rate	0.3	h^{-1}	(4)	0.3	0.3
k_L	Synthesis rate of T7 lysozyme	4500	molecule $\cdot h^{-1} \cdot cell^{-1}$		1900	1900
d_L	Degradation rate of T7 lysozyme	0.0144	h^{-1}	(5)	0.0144	0.0144
T_*	Half activation constant of T7RNAP	1200	molecule $\cdot cell^{-1}$		1276	1276
k_T	Synthesis rate of T7RNAP	6000	molecule $\cdot h^{-1} \cdot cell^{-1}$		8500	8500
d_T	Degradation rate of T7RNAP	0.3	h^{-1}	(5)	0.3	0.3
k_{LT}	Combination rate of T-Lys complex	400	molecule $^{-1} h^{-1} \cdot cell$	(6)	979	979
d_P	Dissociation rate of T-Lys complex	10800	h^{-1}	(6)	10800	10800
k_D	Equilibrium association constant of T7-lysozyme complex	0.037	molecule $^{-1} \cdot cell$	(6)		
P_*	Half inhibition of T-Lys complex	400	molecule $\cdot cell^{-1}$		781	781
α	Inhibition factor of T7RNAP on Growth	1			1	1
β	Inhibition factor of T7 lysozyme on Growth	100			81	
m	Hill coefficient of AHL mediated gene expression	2		(5)	4	4
n_φ	Hill coefficient for distance-dependent	7			7	2

	gene expression capacity					
K_{φ}	Half activation distance for gene expression	0.02	cm		0.02	0.1

Here, k_D is the equilibrium association constant of T7-lysozyme complex, expressed as $\frac{k_{LT}}{d_P}$.

For further details of non-dimension process and numerical value for each parameter, see reference (1) in S1 Text.
